# Diagnostic and Imaging Features of a Non-seminomatous Germ Cell Tumor of the Testis: A Case Report and Review of the Literature

**DOI:** 10.7759/cureus.32305

**Published:** 2022-12-07

**Authors:** Pooja Ladke, Avinash Dhok, Suresh Phatak, Kajal Mitra, Yash Jakhotia

**Affiliations:** 1 Radiodiagnosis, N. K. P. Salve Institute of Medical Sciences & Research Centre and Lata Mangeshkar Hospital, Nagpur, IND

**Keywords:** non-seminomatous germ cell tumor, computed tomography, elastography, germ cell tumor, seminomatous, non-seminomatous

## Abstract

Neoplasms involving the testes are uncommon. They account for up to 10% of all malignant diseases affecting the male genitourinary system and for around 2% of all malignant neoplasms in males. Testicular tumors are the third highest cause of mortality in males between the age of 20 and 40 years. We present a case of a young patient with right-sided scrotal swelling associated with pain. Ultrasonography, elastography, computed tomography, and magnetic resonance imaging features revealed non-seminomatous germ cell tumors of the testis. The patient underwent inguinal orchidectomy for the same and was sent for histopathological evaluation. The imaging diagnosis was consistent with the histopathological diagnosis. This case report adds to the knowledge of imaging features of testicular tumors and their identification based on imaging.

## Introduction

Germ cell tumors, sex cord-stromal tumors, and extragonadal tumors are the three principal testicular neoplasm forms. Seminomas and non-seminomas are the two main kinds of germ cell tumors according to histology [1].

A total of 95% of testicular malignancies in young men are germ cell tumors, and 5% are sex cord-stromal tumors. About half of germ cell malignancies are seminomas, whereas the other half are non-seminomatous germ cell tumors (NSGCTs) [2].

Due to varied presentations, a specific diagnosis of the non-seminomatous mixed germ cell tumor is complex and challenging. Here, we present a rare case of a non-seminomatous mixed germ cell tumor of the testis with yolk sac and embryonal components with emphasis on imaging features.

## Case presentation

Patient and observation

A 35-year-old male patient came with complaints of right-sided scrotal swelling and low-grade scrotal pain for three months. On local examination, the right hemiscrotum was comparatively larger than the left and the right testis was firm in consistency. The left scrotum and testis were grossly normal. The patient denied any medical or surgical history, drug allergy, or trauma. The patient was vitally stable and afebrile with a normal physical examination.

Diagnostic assessment

On laboratory correlation, hematocrit levels were found to be normal. Red blood cell count, white blood cell count, and serum electrolytes were all within normal limits. Beta human chorionic gonadotropin was within normal limits measuring 2.4 IU/L (5 m IU/L), alpha-fetoprotein (AFP) showed increased levels measuring 3.50 ng/ml (0.0-0.9 ng/ml), and lactate dehydrogenase (LDH) showed increased levels measuring 485 IU/L (100-190 IU/L). The radiograph of the chest was normal.

Clinical findings

**Figure 1:**
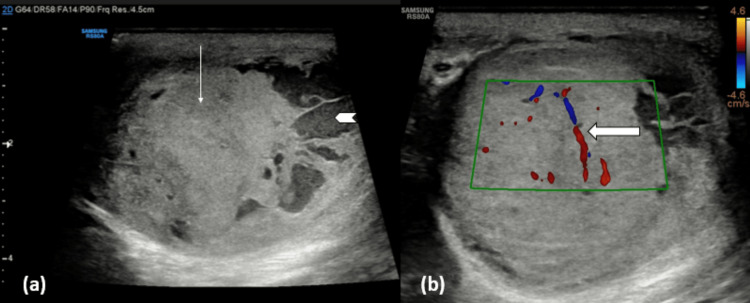
Ultrasonography scrotal image (a) showing a heterogenous lesion with solid (line arrow) and cystic (chevron arrow) components in the right testis and (b) raised vascularity of the testicular lesion (block arrow).

On ultrasound of the scrotum (Figure 1), a relatively well-defined, heterogeneous lesion was noted involving the right testis with solid and cystic components. On color Doppler, the lesion showed increased vascularity.

The left testis was normal in size, shape, echo pattern, and vascularity. Epididymis and spermatic cord were normal on both sides. On scanning the abdomen, no evidence of retroperitoneal lymphadenopathy was seen.

**Figure 2:**
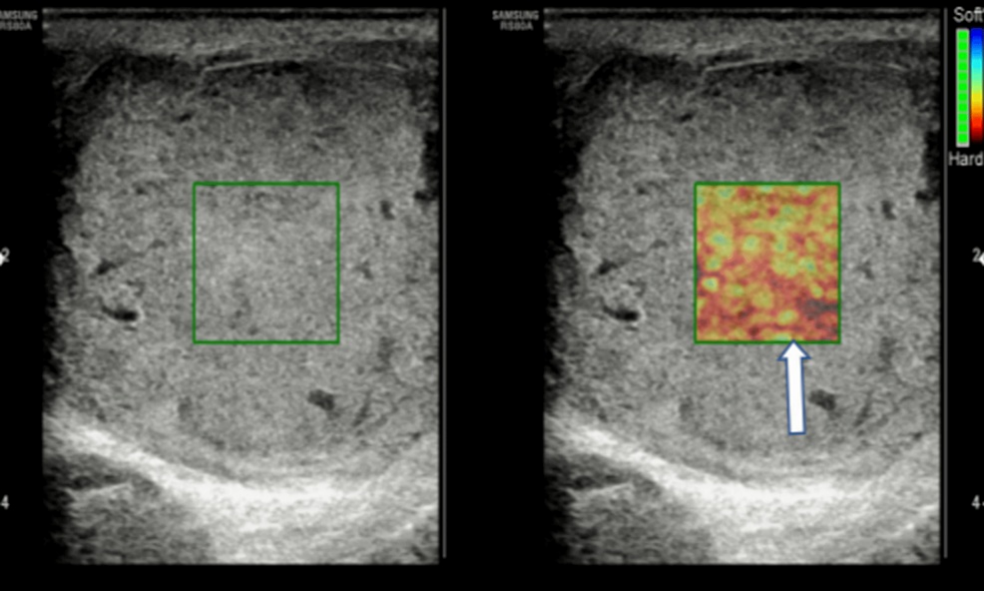
Shear wave elastography image showing hard tissue stiffness, as shown in red (block arrow).

On the shear wave elastography (Figure 2), the lesion demonstrated increased stiffness, shown in red with 50 kPa and a mean velocity of 1.6 m/sec, suggesting a malignant testicular lesion.

**Figure 3:**
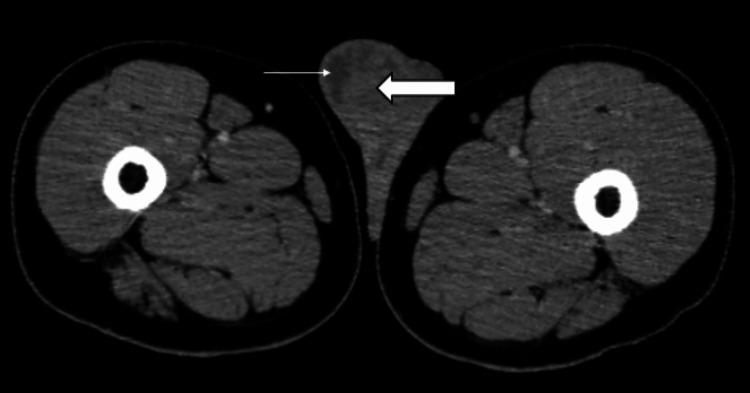
Post-contrast axial CT section at the level of testis showing heterogeneously enhancing right testicular lesion (block arrow) with cystic areas noted within (line arrow).

On contrast-enhanced computed tomography (Figure 3), a partially well-defined heterogeneous density lesion was noted in the right testis with both solid and cystic components. There was no evidence of retroperitoneal or para-aortic lymphadenopathy.

**Figure 4:**
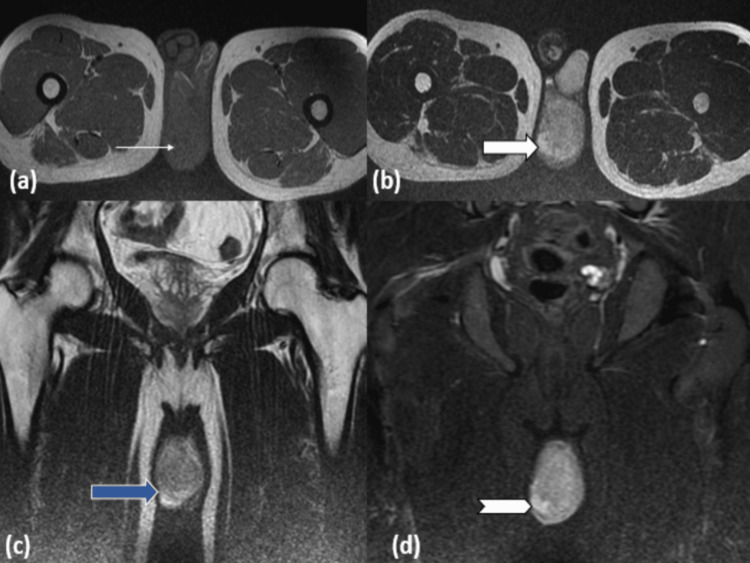
MRI axial T1-weighted image (a) at the level of testis showing iso to hypointense lesion in the right testis (line arrow). Axial (b) and coronal (c) T2-weighted image at the level of testis showing heterogeneously hyperintense lesion in the right testis with cystic spaces (block and blue arrow). Coronal STIR image (d) showing heterogeneously hyperintense lesion in the right testis (chevron arrow). STIR - short tau inversion recovery.

MRI (Figure 4) showed a relatively well-defined heterogeneous iso to hypointense lesion on T1-weighted imaging and a hyperintense lesion on T2-weighted imaging involving the right testis. The lesion on T2-weighted imaging showed multiple cystic spaces, which are suggestive of the necrotic component. The lesions appeared heterogeneously hyperintense on the coronal section of the short tau inversion recovery (STIR) sequence.

Diagnosis

Based on the clinical and imaging features, the provisional diagnosis of a non-seminomatous germ cell tumor of the testis was considered.

Therapeutic intervention

The patient underwent right-sided high inguinal orchidectomy and the tissue was sent for histopathological examination. Histopathologic features were suggestive of a non-seminomatous mixed germ cell tumor with embryonal and yolk sac tumor components. Postoperative chemotherapy was not started, as there was no evidence of the advanced stage of the tumor.

## Discussion

Testicular tumors are categorized mainly into two groups based on their pathology: germ cell and non-germ cell tumors. Germ cell tumors originate from the germinal epithelium and non-germ cell tumors originate from the gonadal stroma. Tumors originating from germ cells comprise most of the testicular tumors, approximately comprising 95% of them. Germ cell tumors are further categorized into two categories: seminomatous, which comprise about 45%, and non-seminomatous tumors, which may be prevalent among the population either in their pure or combined form [3].

Testicular cryptorchidism, familial history, prior history of testicular cancer, and infertility are risk factors for testicular tumors. The variety of non-seminomatous testicular germ cell tumors is hypothesized to result from a totipotent cell line that may differ to varying degrees into embryonal, teratoma, yolk sac, or choriocarcinoma components [4]. Painless enlargement of the testis is the most frequent sign at the time of diagnosis. At the time of the initial diagnosis, 20% of the patients will have metastatic disease and may exhibit abdomen or back discomfort, a general feeling of malaise, or lethargy. This is frequently a sign of retroperitoneal or abdominal lymphadenopathy [5].

Before undergoing any procedure, including orchiectomy, serum tumor markers like AFP, human chorionic gonadotropin, and LDH should be tested since they may be raised in non-seminomatous testicular germ cell tumors [2]. Multiple germ cell components are expressed in mixed germ cell cancers. Rare cases of somatic-type cancer in testicular germ cell tumors also exist. The majority of the time, these components do not primarily affect the testis but rather manifest as metastases, especially after chemotherapy [6].

When testicular malignancy is suspected after a physical examination, an ultrasound of both testes is the primary imaging technique that is advised. Ultrasound of the testis demonstrates usually a solid-cystic heterogenous predominantly hypoechoic intra-testicular lesion with raised vascularity [2]. Shear wave velocity is measured via shear wave elastography to provide a quantitative evaluation. Since non-seminomatous tumors are made up of undifferentiated cells with a significant stromal component, the difference in shear wave velocities between seminomas and non-seminomas is caused by variations in their histologic characteristics [7]. Characterizing the testicular neoplasms is achievable by utilizing T1- and T2-weighted sequencing. A seminomatous lesion is present when there is a homogenous testicular mass with low signal intensity on T2-weighted imaging. On the other hand, a non-seminomatous lesion is defined as a noticeably heterogeneous mass with regions of necrosis or bleeding [8].

Preoperative histologic assessment of different intratesticular masses using MRI of the testicles may produce good findings in terms of morphologic information and demonstrating the presence of fat, fibrous tissue, fluid, and solid tissue inside the masses. Based on MRI findings, a benign lesion diagnosis might lead to better patient care and a reduction in the need for unnecessary surgery [9]. The principal imaging method for evaluating therapy response is still CT. Even if malignant cells remain in the residual tissue, the key change on a CT scan that demonstrates a favorable response to therapy is a reduction in the size of metastases [10].

## Conclusions

Testicular tumors are curable with surgery and chemotherapy. Ultrasound with elastography is used for the initial diagnosis of testicular cancers. CT and MRI aid in the staging of cancer and early detection of metastasis. This case report adds to the knowledge of the significance of radiological imaging in the diagnosis of testicular tumors.
